# Ramadan Fasting and Short-Term Maximal Physical Performance: Searching for Optimal Timing of the Last Meal “Suhoor” in Female Pre-University Handball Players

**DOI:** 10.3390/ejihpe13100152

**Published:** 2023-10-07

**Authors:** Houda Bougrine, Atef Salem, Nidhal Nasser, Achraf Ammar, Hamdi Chtourou, Nizar Souissi

**Affiliations:** 1 Physical Activity Research Unit, Sport and Health (UR18JS01), National Observatory of Sports, Tunis 1003, Tunisia; houdabougrine@live.fr (H.B.); nidhalnasser@issepsf.u-sfax.tn (N.N.); h_chtourou@yahoo.fr (H.C.); n_souissi@yahoo.fr (N.S.); 2 High Institute of Sport and Physical Education Gafsa, Gafsa University, Gafsa 2100, Tunisia; 3 High Institute of Sport and Physical Education Sfax, University of Sfax, Sfax 3000, Tunisia; 4 Department of Training and Movement Science, Institute of Sport Science, Johannes Gutenberg-University Mainz, 55099 Mainz, Germany; 5 Research Laboratory, Molecular Bases of Human Pathology, LR19ES13, Faculty of Medicine of Sfax, University of Sfax, Sfax 3029, Tunisia; 6 Interdisciplinary Laboratory in Neurosciences, Physiology, and Psychology: Physical Activity, Health, and Learning (LINP2), UFR STAPS (Faculty of Sport Sciences), UPL, Paris Nanterre University, 92000 Nanterre, France; 7 High Institute of Sport and Physical Education Ksar-Saïd, Manouba University, Mannouba 2010, Tunisia

**Keywords:** ramadan intermittent fasting, suhoor, nutrient timing, sleep patterns, physical performance, female athletes

## Abstract

Aiming to identify the ideal suhoor timing for maintaining optimal physical performance and health indicators during Ramadan intermittent fasting, the present study compares the effects of early vs. late Suhoor on short-term high-intensity physical exercise while controlling the body mass index (BMI) oral temperature (OT), dietary intake, and sleep patterns. In a randomized design, 19 female pre-university handball players (age: 16.8 ± 0.4 y; height: 1.70 ± 0.9 m; and body mass: 61.5 ± 6.9 kg) underwent two test sessions (at 08:00 a.m. and 05:00 p.m.) at four different conditions: ten days prior to Ramadan (R − 10), the final ten days of Ramadan (R) including both Early Suhoor R(ES) and Late Suhoor R(LS) conditions, and the ten days immediately following Ramadan (R + 10). A recovery period of at least 48 h has been set between successive test sessions at each period. Outcome measures included the Countermovement Jumps Test (CMJ), Modified Agility *t*-Test (MATT), Repeated Sprint Ability (RSA), and Rating of Perceived Exertion (RPE). The Pittsburgh Sleep Quality Index (PSQI), OT, BMI, and daily diary intake were assessed across the three periods. The total scores of PSQI decreased significantly during R and R + 10 compared to R − 10. When performed in the afternoon, CMJ, MATT, and RSA performance decreased significantly at R(ES) and R(LS) conditions compared to R − 10. However, these performances decreased only after R(ES) when performed in the morning. Furthermore, performances were lower during R(ES) compared to R(LS) in the afternoon for all tests and the morning for MATT and RSA tests. These findings support prior research showing a deterioration of physical performance during Ramadan fasting and indicate a more pronounced impact following early Suhoor condition. Therefore, consuming a late suhoor, closer to pre-dawn time, could be suggested as an effective strategy to minimize physical performance decline during short-term high-intensity exercise.

## 1. Introduction

While ball team sports involve diverse technical, physiological, and cognitive performances that are influenced by various factors, there are common objectives related to nutrition in order to perform optimally in major competitions, adapt to training, and minimize injury and illness risks. Specific recommendations can be made to determine the appropriate quality, quantity, and timing of food and drink intake to improve overall well-being and athletic performance in various training and competition contexts. While the relationship between different nutrient intakes and athletic achievement has long intrigued researchers for an extended period, the temporal aspect of nutrient intake is a relatively new area of study that has gained increasing interest in recent years [1,2]. Recent data revealed that the timing, amount, and composition of nutrients can have a major impact on optimizing an athlete’s endurance, strength, and recovery [3,4]. On the other hand, success in competition for limited amounts of food and/or beverages for different periods of time has remained an important issue for athletic performance. 

In sports, Ramadan Intermittent Fasting (RIF) represents one of these conditions, as it is a unique mode of fasting requiring continuous daily fasting of food and drink, including water, for 12 to 18 h (depending on geographical location, the season of the year, and solar calendar dates) over 29–30 days [5]. During Ramadan month, Muslims often eat a couple of meals per day: one at the completion of their daily fast at sunset, known as iftar, and the second before the break of dawn, known as suhoor [5]. The increased demands of modern sports, linked with the rise of the number of Muslim athletes in Western (non-Muslim) countries, have compelled sports experts to investigate the impact of RIF on physical performance within this time frame. Consequently, studies surrounding this topic could aid Muslim athletes and their coaches in strategizing competitions and optimizing their performance during this period.

Indeed, this prolonged fasting from food and fluids (from dawn and sunset), accompanied by a shift in meal timing to the hours of darkness, may affect the biological clock [6], nighttime sleep quality [7], and athletic performance [8]. Evidence about changes in food intake and sleeping habits during this month has incited several researchers to explore the effect of RIF on athletes’ performance, though the evidence remains inconclusive, particularly with regard to short-term high-intensity exercises. In fact, previous findings reported a decrease in short-term high–intensity during RIF in agility and jumping performance [5,9] and repeated sprints [10,11]. Contrary to these studies, some other studies did not observe the negative impacts of RIF on jumping performance [12,13], agility [13,14], or repeated sprints [15]. 

To explain this decline in performance, researchers have also explored the effects of RIF on sleep quality and quantity [5,7] and dairy intake [5,9]. Apart from affecting performance, the daily caloric quantity intake was suggested as a factor contributing to decreased performance during fasting by some studies [16,17]. In contrast, several studies revealed no significant differences in this parameter during the RIF month compared to before Ramadan [5,18]. Meal time, however, has recently been shown to affect energy balance [19,20]. Nevertheless, the caloric restriction during RIF, fewer daily meals (only two meals), and dietary changes may lead to critical daytime dehydration [21], lower total protein synthesis and energy availability [22,23], decrease the availability and use of energy substrates, as well as establish alterations in hormonal and metabolic responses in combination with dehydration [24,25]. Surprisingly, to date, no study has explored the effects of the timing of the last meal (suhoor) before fasting on athletic performance, especially when the fasting hours are prolonged. While research on the timing of meals during RIF is lacking, current data suggests that nutritional factors, such as energy intake, macronutrient composition, and meal timing, could potentially impact athletes’ sleep patterns [26]. Hence, athletic performance could be impaired by a shifted circadian rhythm, altered timing and frequency of meal consumption, reduced calorie and carbohydrate intake, altered metabolic pattern, irregular sleeping duration and timing, and dehydration [22]. Interestingly, calorie restriction has been associated with prolonged sleep onset and reduced slow-wave sleep in overweight women [27]. Given that Suhoor timing is influenced by individual preferences and lifestyles during RIF, and considering that circadian rhythms can impact the body’s response to food and exercise at varying times, it is essential to take these rhythms into account when determining the ideal timing for Suhoor in relation to physical performance.

On the other hand, although half of the sport’s participants are female, less is known about nutrition to promote both health and performance tailored to the unique physiological characteristics of female athletes [28]. While athletes require a well-balanced and sufficient nutrient intake to support their training and performance, the optimal timing of nutrient intake in RIF for athletes is not well-defined and requires further investigation. Although the importance of nutrient timing is increasingly recognized, current recommendations are often based on limited evidence and are not standardized, leading to confusion among athletes and coaches, which may lead to suboptimal performance and an increased risk of injury [29]. Thus, specific nutrient timing guidelines during RIF are needed. 

With this background of evidence, to our knowledge, there is no evidence that the late suhoor intake before starting the fast will have a more significant effect on physical performance. Therefore, the main aim of this investigation was to examine the effect of the timing of the last meal (suhoor) on different aspects of short-term high-intensity performance during morning and afternoon exercises in adolescent female handball players and provide insights into how athletes can optimize Ramadan performance by manipulating their mealtimes. It was hypothesized that (i) RIF would negatively impact short-term high-intensity physical performance primarily in the afternoon rather than the morning, and (ii) late Suhoor intake may mitigate the decline in short-term high-intensity exercise performance caused by RIF when compared to an early Suhoor intake.

## 2. Materials and Methods

### 2.1. Participants 

To allow for an estimated 15% dropout rate at follow-up, 19 pre-university female handball players from the first Tunisian league team were voluntarily involved in this study, whose average age, height, weight, and IMC were 16.8 ± 0.4 years; 1.70 ± 0.9 m; 61.5 ± 6.9 kg; and 21.6 ± 1.8 kg/m^2^, respectively. All players gave their written consent (free and informed) after being informed in detail about the experimental procedures and their constraints before participating, and they could withdraw from the study at any time. Since all our participants are under the age of 18, parental written approval was required. All coaches have also given their official consent. The Local Research Committee granted ethical permission for the protocol and experimental procedure. All the study’s protocols adhere to the 1975 Declaration of Helsinki’s ethical standards [30]. 

They were in and regularly engaged for 2 h a day, 5 days a week, for at least 3 years. Prior to Ramadan, they established a set schedule for eating meals (breakfast at 07:00 a.m. ± 1:00 h, lunch at 12:00 a.m. ± 1:00 h, and dinner at 08:00 p.m. ± 1:00 h) and sleeping (sleeping between 10:30 p.m. ± 1:00 h and 07:00 a.m. ± 1:00 h) according to their daily sleep-wake cycle. In order to guarantee homogeneity and reduce the dispersion of circadian topology in the experimental populations, the players were selected according to their answers to the Horne and Ostberg [31] self-evaluation questionnaire of the type of sleep, which rates morningness and eveningness. Based on their answers to questions, subjects who showed an extreme morning or evening type were disqualified. Instead, all investigation participants were chronotypes of the “neither type”, with scores on the scale ranging from 46 to 57. 

All athletes had a typical sleep duration of 7.8 ± 0.4 h in the month prior to the experimental method, as measured by the Pittsburgh Sleep Quality Index (PSQI) [32]. Moreover, to ensure consistency and minimize the influence of the habitual timing of suhoor among the participants in the study, subjects were chosen based on their responses to the daily time of suhoor during the first 20 days of RIF. Participants who presented extreme habitual times of suhoor (consumed at ≤10:30 ± 30 p.m. or ≥03:30 ± 30 a.m.) that coincided with our two chosen times in our study were disqualified. Therefore, the study included players with daily habitual timing suhoor at 01:30 ± 60 a.m. The inclusion criteria were as follows: (i) no history of major medical conditions, smoking, caffeine dependence, or daily habitual napping; (ii) observing Ramadan for at least three years; (iii) not using any form of contraception, including pills, patches, injections, implants, or intrauterine devices; and/or had any menstrual or endocrine abnormalities in the previous six months. All phases of the menstrual cycle of participants were assessed during all testing sessions using a mobile application (MyCalendar^®^; Period Tracker) that identifies the main events taking place throughout the menstruation cycle [33]. “Moreover, only players who had not been previously affected by COVID-19 were recruited. This decision was made due to concerns that the COVID-19 pandemic might have effects on athletes’ physical activity, including lower sleep quality than recommended and higher insomnia symptoms, as well as on their mental health and quality of life, such as a mismatch between circadian and social clocks pre-lockdown [34]. 

### 2.2. Tool

For the experimental sessions, players reported to the course one hour before the start of sessions for data collection, where the pre-experimental assessments, basic anthropometry, and experimental trials were performed. During each experimental session, oral temperature and anthropometric measurements were recorded at the beginning of each test session. Body mass was measured using an electronic scale (Tanita, Tokyo, Japan). A digital clinical thermometer (Omron, Paris, France; accuracy +0.05 °C) was inserted sublingually for at least three minutes after a 10-min period of resting while seated in order to measure the oral temperature. Following that, athletes performed a standardized warm-up (~10 min including rest), which consisted of 3 min jogging (at 8–10 km·h^−1^). Jogging was followed by 3 min of whole-body dynamic stretching, 2 min of sprint drills (ankling, high-knee, backkick, and skipping), and 2 min of sprinting. Athletes then performed during each test session in the same order: Countermovement Jumps test (CMJ), Modified Agility *t*-Test (MATT), Repeated Sprint Ability (RSA), and Rating of Perceived Exertion (RPE). A 5-min rest was taken between tests to ensure appropriate recovery. Moreover, the RPE was carried out by having players respond verbally after each 25 m shuttle sprint in the RSA test.

Testing sessions were conducted at an indoor training facility with relatively similar ambient temperatures (~25 °C, 28 °C, and 28 °C) and relative humidity (~54%, 48%, and 49%) during the three experimental periods of our study, R − 10, R, and R + 10, respectively. The study was conducted in Tunisia, where Ramadan took place on 2 April and ended on 1 May 2022.

During this study, the periods of daily fasting were as follows: from 04:25–03:28 a.m. to 06:43–07:10 p.m. local time (approximately 14.5–15.5 h). When our two suhoor conditions were evaluated during the last ten days of Ramadan, the daily fasting times were as follows: from 03:54–03:38 a.m. to 07:01–07:10 p.m. local time (about 15–15.5 h).

Throughout the experimental period, participants were requested to maintain their habitual physical activity and avoid strenuous activities or consuming any caffeine the day before each session. Additionally, athletes were instructed to reproduce the first day’s diet at each experimental trial and to standardize their meals for the 24 h before each test. During the last ten days of Ramadan, all athletes adhered to the following lifestyles: (a) Their sleep schedule consisted of several short intervals, specifically from approximately 11:30 p.m. to 1:30 a.m., 2:30 a.m. to 7:00 a.m., and 2:30 p.m. to 4:00 p.m. (b) They consumed two meals: the first at iftar between 7:00 p.m. and 7:30 p.m., and the second between 10:30 p.m. and 11:30 p.m. on days 22 and 24 to ensure an early Suhour; between 1:30 a.m. and 2:30 a.m. on days 26 and 28 to ensure a late Suhour; or at one of these two time slots in the remaining days. (c) The athletes engaged in only one daily training session, which took place from 9:00 p.m. to 10:00 p.m. During the testing trials, only tactical and technical training sessions were allowed in order to prevent strenuous activities that could impact our outcomes.

#### 2.2.1. The Pittsburgh Sleep Quality Index (PSQI) 

As described by Bougrine et al. [35], the validated Arabic version of the Pittsburgh Sleep Quality Index [32] was used to assess the subjective quality of sleep throughout the course of the previous month and for the duration of each experimental test period (10 consecutive days). 

#### 2.2.2. Dietary Intake Analysis 

As described by Bougrine et al. [35], participants recorded their food intake in a food consumption diary throughout the span of a 10-day period during each of the three testing periods (R − 10, R, and R + 10). 

#### 2.2.3. Countermovement Jump Test 

The participants executed a quick vertical jump with downward eccentric and upward concentric moves while standing upright. They were instructed to jump as high as they could and land in the same spot while keeping both hands on their hips to minimize the impact of the arm swing. The Microgate software (Optojump software, version 1.10.50) and the Optojump-next device (Bolzano, Italy) were both used. For analysis, only the jump height (cm) was recorded. Throughout the test, each participant completed three trials, separated by a two-minute recovery period, and the highest jump achieved among these three trials was recorded for subsequent analysis.

#### 2.2.4. Modified Agility *t*-Test (MATT) 

The modified agility *t*-test involved multidirectional sprinting, shuffling, and backpedaling [36,37]. A timing gate (Witty, Microgate^®^, Bolzano, Italy) was used to record time at the start/finish line (the same line for this test), while athletes began the test 0.5 m behind the gate. Participants began with a 5 m linear sprint to cone B, followed by a 2.5 m leftward shuffle to cone C, a 5 m rightward shuffle to cone D, a 2.5 m leftward shuffle back to cone B, and a 5 m linear backpedal to cone A. For each trial, the total distance covered was 20 m. The MAT performance score was the fastest time from two trials, interspersed with three minutes of rest.

#### 2.2.5. Repeated Sprint Ability (RSA) 

Similar to previous studies on female athletes [9,38,39], repeated sprint ability (RSA) testing involved six maximal 2 × 12.5 m shuttle sprints, with 20 s of passive recovery between sprints and 180° turns. The RSA was established to measure repeated sprints with a change in direction. The timing gates (Witty, Microgate^®^, Bolzano, Italy) were used to record the times. Athletes were verbally motivated during testing and instructed to get into the starting position 0.5 m behind the starting line 6 seconds prior to the start of each sprint until the next start signal. The digital timer started automatically when the photocell beam was disrupted. A light panel (Microgate^®^, Bolzano, Italy) was then used to visually give participants a countdown of 3 s. They sprinted for 12.5 m from the starting line, touched the second line with one foot, and then returned to the starting line with as much speed as possible. Participants were given instructions to complete all sprints as quickly as possible. For analysis, the best sprint (RSAbest) and the mean time of all sprints (RSAmean) were retained.

#### 2.2.6. Rating of Perceived Exertion (RPE)

The RPE scale is a valid predictor of physical discomfort, has high psychometric qualities, and has a significant correlation with various other physiological exertion measurements. The score on the [40] 10-point scale runs from 0 (nothing; represents how you feel when sitting in a chair) to 10 (very, very heavy; represents how you feel after performing a very, very hard exercise).

### 2.3. Procedure 

**Figure 1:**
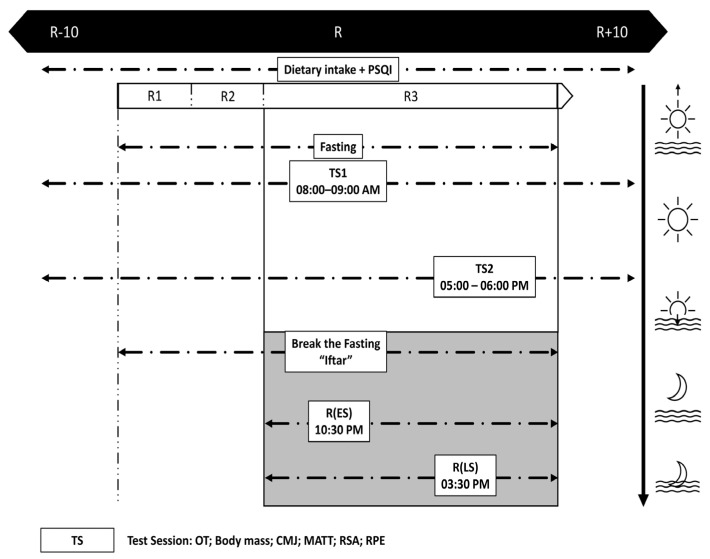
Study design. R − 10: the 10 days before Ramadan, R: Ramadan, R1: First 10 days of Ramadan, R2: Second 10 days of Ramadan, R3: Last 10 days of Ramadan, R + 10: the 10 days before Ramadan, R(ES): Early Suhoor, R(LS): Late Suhoor, PSQI: Pittsburgh Sleep Quality Index, OT: Oral temperature, CMJ: countermovement jump test, MATT: Modified agility *t* test, RSA: Repeated sprint ability test, RPE: Rating of perceived exertion; all times given are expressed in local time (GMT  +  1  h).

The experimental design is displayed in Figure 1. In order to reduce learning effects during the experiment and guarantee high-quality results, all players were familiarized with the experimental protocol and the equipment throughout the course of the two weeks before the study at 08:00 a.m. and 05:00 p.m. 

This study followed a randomized counterbalanced crossover trial where all participants were tested under four different conditions: the ten days prior to Ramadan (R − 10), the final ten days of Ramadan (R), including two conditions: Early Suhoor R(LS) at day 22 and 24 of Ramadan and Late Suhoor R(LS) at days 26 and 28 of Ramadan, and the ten days immediately following Ramadan (R + 10). Athletes completed two counterbalanced test trials throughout each condition, with only one test session per day, one in the morning (08:00–09:00 a.m.) and one in the afternoon (05:00–06:00 p.m.), separated by a minimum of 48 h. These two times of day were chosen because they roughly refer to the batyphase and acrophase of maximum short-term performance and oral temperature [41] (Figure 1). 

#### Suhoor Protocol 

During the Ramadan period, players randomly achieved two conditions concerning the timing of suhoor: R(ES): Early Suhoor (consumed at 10:30 p.m.) and R(LS): Late Suhoor (consumed at 03:30 a.m.). All athletes received the same standard suhoor, with the caloric intake set at 650–700 kcal (60% carbohydrates, 20% protein, and 20% fat) with two glasses of water (225 mL per glass), based on the results of the food diary checked and evaluated by the same dietician before and throughout Ramadan. In terms of macronutrient intake, the suhoor quality and quantity did not differ much from the athlete’s baseline routine; however, the two conditions differed in the time at which they had their meals. It’s worth noting that athletes were under continuous surveillance during both different experimental conditions and that the ingestion was verified by an experimenter. Subjects ate their last meal at least 20 min before going to bed. No instructions for sleeping time were given to participants after the suhoor; however, our instructions were limited to the timing of the suhoor. No food and/or beverage was allowed for participants after taking the standard suhoor meal. 

### 2.4. Statistical Analysis 

The required sample size was determined a priori using the G*Power 3.1 software [42]. The values for α and power were set to 0.05 and 0.95, respectively. Effect sizes have been estimated to be 0.39 based on studies with identical methodologies [14,43] and discussions among the authors. To reach the desired power, data from at least 16 participants was considered sufficient to minimize the risk of incurring the probability of a type 2 statistical error. 

All statistical tests were processed with STATISTICA 10 software (StatSoft, Paris, France). Means ± SD (standard deviation) values were calculated for each variable. All data were normally distributed, as confirmed using the Shapiro–Wilk test. For the effect of time of suhoor, a two-way repeated measures ANOVA [4 (Testing conditions) × 2 (Time of the day)] was conducted. Where appropriate, significant differences between means were tested using Tukey’s HSD Post hoc test. A one-way repeated measures ANOVA (3 testing periods) was used to analyze the following data: PSQI, body mass, BMI, energy intake, fat (g), carbohydrate (g), and protein (g). When significant differences were reported, Tukey’s HSD Post hoc test was used to test between means. The magnitude of the difference between age groups was evaluated using the effect size statistic (η^p2^). The criteria used to determine the effect sizes were as follows: 0.01 denoted a small effect size, 0.06 represented a moderate effect size, and 0.14 indicated a large effect size [44]. Standardized effect size (Cohen’s d) analysis was used to interpret the magnitude of differences between variables and classified according to [45] as trivial (d ≤ 0.20); small (0.20 < d ≤ 0.60); moderate (0.60 < d ≤ 1.20); large (1.20 < d ≤ 2.0); very large (2.0 < d ≤ 4.0); and extremely large (d > 4.0). A significant level was considered as *p* ≤ 0.05.

## 3. Results

### 3.1. The Pittsburgh Sleep Quality Index (PSQI)

The one-way ANOVA test demonstrated significant main effects of conditions on sleep latency (F (2, 36) = 4.12, *p <* 0.05, η_p_^2^ = 0.18), sleep duration (F (2, 36) = 53.69, *p <* 0.001, η_p_^2^ = 0.74), sleep quality (F (2, 36) = 88.98, *p <* 0.001, η_p_^2^ = 0.83), sleep disturbances (F (2, 36) = 36.29, *p <* 0.001, η_p_^2^ = 0.66), daytime dysfunction (F (2, 36) = 27.76, *p <* 0.001, η_p_^2^ = 0.60), and total scores of PSQI (F (2, 36) = 66.73, *p <* 0.001, η_p_^2^ = 0.78), while sleep efficiency and the use of sleeping medication remained unaffected. The Tukey test revealed the following significant findings: (i) Sleep latency was longer during R compared to R − 10 (*p <* 0.05), (ii) Sleep duration decreased significantly during R and R + 10 (both *p <* 0.001) compared to R − 10, (iii) The total scores of PSQI, sleep quality, and sleep disturbance scores increased significantly during R and R + 10 (all *p <* 0.001) compared to R − 10, and (iiii) The daytime dysfunction score was higher during R (*p <* 0.001) and R + 10 (*p <* 0.05) compared to R − 10 (Table 1). 

### 3.2. Dietary Intake Analysis, Body mass, and BMI

The analysis revealed no significant statistical differences in the daily mean energy and macronutrient intake, body mass, or BMI among the participants across the three testing phases (all *p* > 0.05; Table 2).

### 3.3. Oral Temperature (OT)

**Figure 2:**
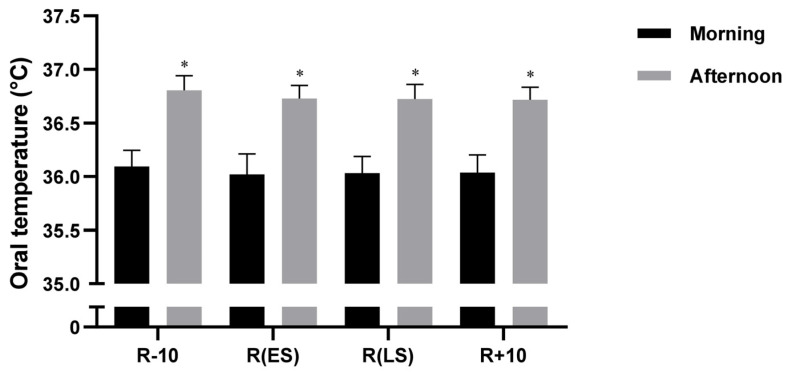
Mean ± SD values of Oral Temperature (OT) measured at 08:00 a.m. and 05:00 p.m. during 10 days before Ramadan R − 10, Ramadan Early Suhoor R(ES), Ramadan Late Suhoor R(LS), and 10 days following Ramadan R + 10. * (*p* < 0.001): Significant difference compared to 05:00 p.m.

Statistical analysis of OT revealed significant main effects of Conditions (F (3, 54) = 6, *p <* 0.01, η_p_^2^ = 0.24) and Time-of-day (F (1, 18) = 468, *p <* 0.001, η_p_^2^ = 0.96). However, there was no significant Conditions × Time-of-day interaction (F (3, 54) = 0, *p* = 0.92, η_p_^2^ = 0.008). Post hoc analysis indicated that OT values were higher in the afternoon compared to the morning in all three testing conditions (all *p* <0.001) (Figure 2).

### 3.4. CMJ

**Figure 3:**
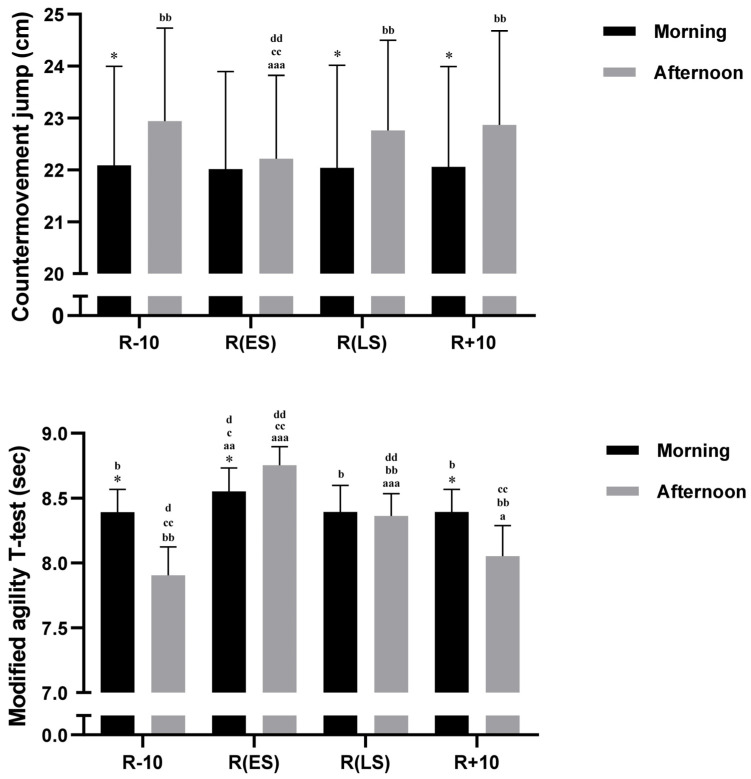
Mean ± SD values of Countermovement Jump Test (CMJ) and Modified Agility *t*-Test (MATT) performances recorded at 08:00 a.m. and 05:00 p.m. during 10 days before Ramadan R − 10, Ramadan Early Suhoor R(ES), Ramadan Late Suhoor R(LS), and 10 days following Ramadan R + 10. * Significant difference compared to 08:00 a.m. (*p* < 0.001). ^a^ (*p* < 0.05), ^aa^ (*p* < 0.01), and ^aaa^ (*p* < 0.001): Significant difference compared to R − 10 at the same time of day. ^b^ (*p* < 0.01) and ^bb^ (*p* < 0.001): Significant difference compared to R(ES) at the same time of day. ^c^ (*p*< 0.01), ^cc^ (*p* < 0.001) Significant difference compared to R(LS) at the same time of day. ^d^ (*p* < 0.01), ^dd^ (*p* < 0.001) significant difference compared to R + 10 at the same time of day.

The two-way ANOVA showed significant main effects of Time-of-day (F (1, 18) = 11.55, *p <* 0.01, η_p_^2^ = 0.39) and Conditions (F (3, 54) = 26.29, *p <* 0.001, η_p_^2^ = 0.59). Additionally, a significant Conditions × Time-of-day interaction was found (F (3, 54) = 12.44, *p <* 0.001, η_p_^2^ = 0.40). The post hoc test demonstrated that during R − 10, R(LS), and R + 10, CMJ performances were higher in the afternoon than in the morning (all *p* < 0.001) (Figure 3). These findings revealed that CMJ performance was reduced in the afternoon compared to R − 10 throughout the R (ES) (*p* < 0.001) (−3, 2%). Additionally, CMJ was greater during R(LS) than R(ES) only in the afternoon (*p* < 0.001) (Figure 3). 

### 3.5. MATT

There were a significant main effects of Time-of-day (F (1, 18) = 14.95, *p <* 0.01, η_p_^2^ = 0.45) and Conditions (F (3, 54) = 143.14, *p <* 0.001, η_p_^2^= 0.88), also a significant Conditions × Time-of-day interaction (F (3, 54) = 58.94, *p <* 0.001, η_p_^2^= 0.760), where MATT performance was better in the afternoon than in the morning during R − 10 and R + 10 (both *p* < 0.001) (Figure 3). In comparison with R − 10, MATT decreased during R(ES) (10, 7%) and R(LS) (5, 8%) and R + 10) (1, 9%) in the afternoon (*p* < 0.001, *p* < 0.001, *p* < 0.05, respectively) and only during R(ES) in the morning (*p* < 0.01) (1, 9%). Moreover, MATT performance was lower during R(ES) compared to R(LS) in the afternoon (*p* < 0.001) and in the morning (*p >* 0.01) (Figure 3). 

### 3.6. RSA 

#### 3.6.1. RSA_mean_

**Figure 4:**
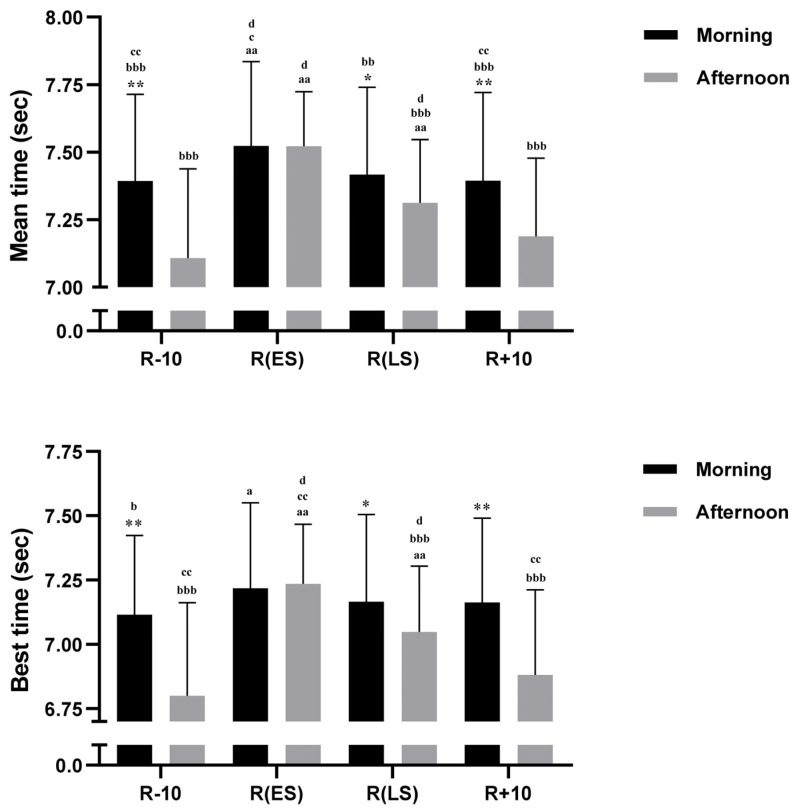
Mean ± SD values of Repeated Sprint Ability (RSA) measured at 08:00 a.m. and 05:00 p.m. during 10 days before Ramadan R − 10, Ramadan Early Suhoor R(ES), Ramadan Late Suhoor R(LS), and 10 days following Ramadan R + 10. *, ** Significant difference compared to 08:00 a.m. (*p* < 0.01, and *p* < 0.001, respectively). ^a^ (*p* < 0.05) and ^aa^ (*p* < 0.001) Significant difference compared to R − 10 at the same time of day. ^b^ (*p* < 0.05), ^bb^ (*p* < 0.01), and ^bbb^ (*p* < 0.001) Significant difference compared to R(ES) at the same time of day. ^c^ (*p* < 0.01) and ^cc^ (*p* < 0.001) Significant difference compared to R(LS) at the same time of day. ^d^ Significant difference compared to R + 10 at the same time of day (*p* < 0.001).

There was a significant effect of Conditions (F (3, 54) = 72.14, *p <* 0.001, η_p_^2^ = 0.80), Time-of-day (F (1, 18) = 9.06, *p <* 0.01, η_p_^2^ = 0.33), and Conditions × Time-of-day interaction (F (3, 54) = 22.39, *p <* 0.001, η_p_^2^ = 0.55). RSA_mean_ performance was better in the afternoon than in the morning during R − 10, R(LS), and R + 10 (*p* < 0.001, *p* < 0.01, *p* < 0.001, respectively) (Figure 4). In comparison with R − 10, RSA_mean_ performance decreased during R(ES) (5, 8%) and R(LS) (2, 9%) in the afternoon (both *p*< 0.001), but only during R(ES) in the morning (*p* < 0.001) (1, 8%). Furthermore, RSA_mean_ was notably lower during R(ES) when compared to R(LS) in both the afternoon (*p* < 0.001) and the morning (*p* < 0.01) (Figure 4). 

#### 3.6.2. RSA_best_

There was a significant effect of Conditions (F (3, 54) = 84.90, *p <* 0.001, η_p_^2^ = 0.82), Time-of-day (F (1, 18) = 9.19, *p <* 0.01, η_p_^2^ = 0.33), and Conditions × Time-of-day interaction (F (3, 54) = 29.02, *p <* 0.001, η_p_^2^ = 0.61). The post hoc test revealed that RSA_best_ performance was higher in the afternoon than in the morning during R − 10, R(LS), and R + 10 (*p* < 0.001, *p* < 0.01, *p* < 0.001, respectively) (Figure 4). Compared to R − 10, RSA_best_ performance decreased during R(ES) (6, 4%) and R(LS) (3, 6%) in the afternoon (both *p* < 0.001) and only during R(ES) in the morning (*p* < 0.05) (1, 4%). Likewise, compared to R(ES), RSA_best_ was better during R(LS) only in the afternoon (*p* < 0.001) (Figure 4).

### 3.7. RPE

**Figure 5:**
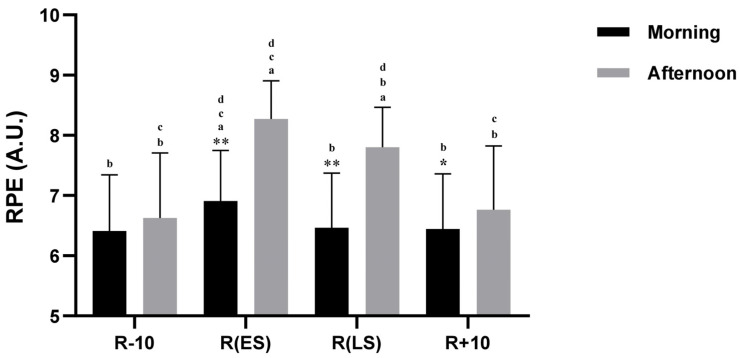
Mean ± SD Scores of Rating of perceived exertion (RPE) recorded at 08:00 a.m. and 05:00 p.m. during 10 days before Ramadan R − 10, Ramadan Early Suhoor R(ES), Ramadan Late Suhoor R(LS), and 10 days following Ramadan R + 10. *, ** Significant difference compared to 08:00 a.m. (*p* < 0.05 and *p* < 0.001, respectively). ^a^ Significant difference compared to R − 10 at the same time of day (*p* < 0.001). ^b^ Significant difference compared to R(ES) at the same time of day (*p* < 0.001). ^c^ Significant difference compared to R(LS) at the same time of day (*p* < 0.001). ^d^ significant difference compared to R + 10 at the same time of day (*p* < 0.001).

The RPE scores showed significant main effects for both Conditions (F (3, 54) = 106.90, *p <* 0.001, η_p_^2^ = 0.85) and Time-of-day (F (1, 18) = 37.96, *p <* 0.001, η_p_^2^ = 0.67). Additionally, there was a significant interaction between Conditions × Time-of-day (F (3, 54) = 42.46, *p <* 0.001, η_p_^2^ = 0.70). The post hoc analysis revealed that RPE scores were significantly higher in the afternoon than in the morning during R(ES), R(LS), and R + 10 (*p* < 0.001, *p* < 0.001, *p* < 0.05, respectively). However, no significant difference was observed during R − 10 (*p* > 0.05) (Figure 5). RPE scores increased significantly during R(ES) (24, 9%) and R(LS) (17, 7%) in the afternoon (both *p* < 0.001) but only during R(ES) in the morning (*p <* 0.001) (7, 8%) compared to (R − 10). When compared to R(LS), RPE scores were higher during R(ES) in both the afternoon (*p* < 0.001) and in the morning (*p* < 0.001) (Figure 5). 

## 4. Discussion

The aim of the present study was to investigate the effect of suhoor timing during RIF on short-term high-intensity physical performance in adolescent female handball players. The main hypotheses formulated were that (i) RIF would negatively impact short-term high-intensity physical performance primarily in the afternoon rather than the morning, and (ii) acute late Suhoor intake may mitigate the decline in short-term high-intensity exercise performance caused by RIF when compared to an early Suhoor intake. The results of our study supported these hypotheses and indicated that (i) RIF significantly impairs short-term high-intensity physical performances and sleep parameters; (ii) The timing of suhoor consumed by athletes observing the fast during Ramadan affects short-term high-intensity exercise; (iii) The acute late suhoor could minimize the physical performance drop during the afternoon period of RIF and maintain the same morning performance, better than early suhoor consumption. 

### 4.1. Effect of RIF

Our PSQI scores demonstrated that RIF substantially impairs sleep duration, with a shift of 90 min compared to before RIF. This shift was accompanied by an increase in the overall PSQI score (higher more than three times) and sleep quality (higher more than four times) compared to before RIF. Whereas recent studies [5,35,43] have highlighted a negative impact on subjective sleep quality during RIF, other studies revealed no significant change in sleep quality or duration [46,47]. The inconsistencies in the effects of RIF on sleep may be due to variations in methodology, study populations, or lifestyle differences. Therefore, recently, it has been shown that the exclusive nighttime ingestion of enormous quantities of food throughout this month, coupled with several lifestyle changes, can diminish sleep quality and duration [7]. The total sleep quantity and quality and the timing of the sleep constitute essential factors to facilitate recovery and achieve optimal athletic performance [48]. This could explain the drop in performance observed during this period of RIF. In the same vein, [5,35] suggested that the cumulative fatigue caused by the repetitive partial sleep deprivation during RIF was the origin of the drop in physical performance and a change in diurnal variation of these performances, not the diary intake. RIF may disrupt sleep patterns, and inadequate or disrupted sleep can negatively affect cognitive function and physical performance [5]. Indeed, it has been shown that insufficient sleep can lead to reduced attention and slower reaction times, particularly in the late afternoon [49,50]. It’s essential to acknowledge that getting less than the recommended eight hours of sleep can result in cognitive performance deficits [51]. Hence, the significance of mental well-being and cognitive abilities in achieving success in sports is increasingly acknowledged, and this awareness extends to team ball players [52].

On the other hand, our findings indicated that RIF could indeed have a negative impact on physical performance in athletes, particularly during the afternoon hours in both conditions (RE) and (RL) of suhoor rather than the morning compared to before Ramadan and immediately following RIF. These findings are in line with recent studies that examined the diurnal variation of short-term maximal exercise during RIF, with a performance drop observed only during the afternoon sessions for jump performance [5,9], agility [9,35], and repeated sprint bouts [5,35]. Other studies, however, found no effect of RIF on squat jump performance [53,54], agility [14], or repeated shuttle tests [10,15]. Methodological variables such as fasting variables (including duration of fast, suhoor time, period of RIF, and the season of fast), age, gender, chronotypes, and physical level of participants could explain discrepancies between studies.

Regardless of the suhoor timing, this drop in performance observed mainly in the afternoon is primarily due to the fasting duration (about 15.5 h during our study). In fact, due to the prolonged duration of fasting during Ramadan (13.5 h during afternoon sessions for R (LS) and 18.5h during afternoon sessions for R (ES)), athletes may experience decreased glycogen stores, lower blood glucose levels after more than 6h of the last meal [55], and overall reduced energy availability, leading to fatigue and decreased performance [56]. Furthermore, the lack of fluid intake during the day can lead to dehydration, which can impair an athlete’s ability to maintain optimal performance levels during physical activities, especially during the hot months [57]. Dehydration can impair cardiovascular function, thermoregulation, and muscular performance, negatively impacting physical performance [8]. However, it should be noted that RIF has also been linked to disturbances in the body’s natural sleep-wake patterns, as well as alterations in the levels of various hormones, including leptin, adiponectin, ghrelin, cortisol, and melatonin [58,59,60]. These physiological changes have been observed during RIF due to abrupt shifts in meal times, modifications in dietary choices, variations in food group intake, and disruptions in sleep patterns, all of which have the potential to disrupt the metabolic effects of RIF [61,62]. All these modifications were linked with an advance or a delay in circadian rhythm phases [63], stress, anxiety, or concerns about fasting that may affect an athlete’s mindset and motivation, potentially impacting their performance [47,64], and deterioration of sleep quality and duration [7]. Furthermore, our findings regarding body mass and BMI align with a recent systematic review [65], which affirmed the lack of substantial evidence supporting a decrease in body composition indicators among athletes during Ramadan. Hence, it has been recently demonstrated that an increase in muscular hypertrophy and the accompanying rise in body weight among athletes, along with a reduction in adipose tissue levels, were considered favorable factors contributing to the enhancement of motor potential [66]. This led us to hypothesize that the lack of motor potential observed during afternoon sessions may be attributed to sleep disturbances and/or the timing of the suhoor meal, not the amount of caloric intake. Furthermore, compared to non-Ramadan time, inadequate sleep and feelings of malaise increase fatigue and perceived effort in response to the same exercise load [67,68]. Further, studies conducted during non-RIF periods indicated that dietary timing, quantity, and quality influenced both sleep duration and quality [69,70]. As a result, it is crucial to investigate the link between sleep quality and caloric intake timing. 

### 4.2. Effect of Time of the Day

The present study indicated that the short-term high-intensity physical performance of female athletes is time-dependent, which suggests that the timing of physical exercise may have an impact on performance outcomes. This finding implies that athletes may experience variations in their performance levels depending on the time of day when they engage in physical activities, with better performances recorded in the afternoon before the Ramadan period. Our results are in line with a recent meta-analysis [71] that demonstrated that late afternoon and early evening (between 04:00 p.m. and 07:30 p.m.) tend to be optimal times for short-term high-intensity physical performance due to numerous factors such as increased core body temperature [72], improved muscle function [73,74], increased hormone levels [74,75] and enhanced cognitive functions such as reaction times [76]. These factors can lead to better jumping abilities [5,77], agility [9,77], and repeated shuttles [5,9,77] during physical exercises in the afternoon for female handball players. However, it is worth noting that other studies have not found a significant difference in performance based on the time of day in several aspects of short-term maximal exercise [78,79]. Factors such as individual variations in chronotype (whether one is a “morning player” or “evening player”, but in our study, it is “neither” chronotype), personal preferences and/or time from wake-up [80], training status and schedules [41], specific sport requirements, and differences in testing protocols can influence the outcomes. Additionally, our results indicated that the diurnal variations of short-term high-intensity physical performance are Ramadan-dependent. The impact of the time of day on physical performance appears to diminish during Ramadan due to a decline in performance observed in the afternoon. These daily fluctuations disappeared, blunted, and/or reversed during this month, which is in agreement with those of [5,35,43].

### 4.3. Effect of Suhoor Timing

Regarding the effect of suhoor timing, an important finding of our study is that there was a significant effect of the timing of suhoor in short-term high-intensity exercise. We found that a late suhoor intake may help to (i) enhance the decline observed in these performances in the afternoon during RIF and (ii) maintain morning performance values similar to those of before Ramadan at the same time of day. We speculate that one possible explanation for the decrease in performance in both conditions of suhoor compared to baseline values, particularly in the afternoon, is that refraining from eating and drinking for extended periods prior to and during physical exercise can result in reduced accessibility and utilization of energy sources, as well as alterations in hormonal and metabolic responses that occur simultaneously with dehydration [24,25]. In fact, this prolonged fasting period can result in various metabolic adaptations that can affect physical performance by reducing total protein synthesis [22] and creating low energy availability [23]. Recently, it has been shown that lipids and hormones were also affected by meal timing rather than glucose levels during time-restricted feeding [81]. Given that glucose is the primary energy source for humans and that glucose metabolism is time-dependent (meaning it depends on how long it has been since the last meal), blood glucose levels drop rapidly after ingesting a carbohydrate meal. Depending on the amount of glycogen stored in the liver and the subsequent energy expenditure, glycogen levels will be lowered, and fat metabolism will become the energy source via ketone body synthesis over the 12- to 36-hour period following carbohydrate intake [82]. Consequently, a decline in blood glucose levels could alter hormone levels, leading to symptoms such as fatigue (which could explain the higher afternoon RPE scores, especially after early suhoor condition), dizziness, hunger, weakness, and a decrease in overall athletic performance [83]. In the morning, athletes typically have the opportunity to consume a pre-dawn meal (suhoor) to provide some fuel and hydration for the day ahead. However, as the day progresses and the fasting period extends, these stored energy reserves gradually deplete. This phenomenon can be possibly explained by the difference in the timing of the “suhoor effect” between the afternoon fasting duration (around 15.5 h vs. 20.5 h) and the morning fasting duration (around 4.5 h vs. 9.5 h) in the LS condition and ES condition, respectively. Only morning performance with late suhoor intake (LS condition) remained unchanged because the fasting period was under 8 h (around 4.5 h). However, the most pronounced declines in performances were recorded in the afternoon under ES condition, where the fasting duration extended to around 20.5 h.

Although research on the timing of meals during RIF is lacking and to the best of our knowledge, current data indicates that meal timing may have the potential to affect athletes’ sleep patterns [26]. In agreement with our findings, calorie restriction has been associated with prolonged sleep onset and reduced slow-wave sleep in overweight women [27]. A recent study revealed that consuming more sugar in the evening and having a longer gap between dinner and bedtime was linked to shorter total sleep duration [84]. Furthermore, it has been demonstrated that minor adjustments in sleep-wake cycles and meal schedules could potentially affect the overall performance patterns in short-distance running [85]. Our results are in line with previous studies that suggested that late suhoor ingestion can help optimize hydration levels before the start of the fast, which is crucial for maintaining optimal cardiovascular function, thermoregulation, and muscular performance [86,87]. In addition, Chtourou et al. [88] also supposed that morning performance was not negatively affected by fasting when athletes consumed a meal before sunrise, and Mhenni et al. [9] suggested that consuming suhoor meal as late as permissible could be the origin of the improvement in physical performance observed during Ramadan in the morning. Unfortunately, the authors did not find any prior studies that had investigated the timing of suhoor (pre-dawn meal) among athletes for comparison with our study results, although it has been recently revealed that reducing or limiting energy intake during a time-restricted feeding for 8 to 12 h causes the body to shift from glucose to fat for fuel [81]. Moreover, carbohydrate intake during exercise assists in carbohydrate oxidation, minimizes hypoglycemia, and has beneficial impacts on the central nervous system [23,81]. Given that nutritional timing is important when compared to breakfast, isocaloric meals with the same nutritional composition seem to provide additional calories when ingested in the evening. This could suggest that, in addition to what athletes decide to consume, when they consume also affects their physiological response to food and postprandial glucose levels [23]. In conclusion, the drop in physical performance during the afternoon of Ramadan can be influenced by factors such as fasting duration, dehydration, energy and nutrient deficits, the timing of the last meal of suhoor, circadian rhythms, sleep disturbances, and psychological factors.

### 4.4. Strength and Limitations

To our knowledge, our study is one of the pioneers’ attempts to examine the relationship between last-meal suhoor timing and short-term high-intensity performance among athletes, based on a study of the diurnal variation of these performances in female athletes. Some limitations of the present study should be taken into account. We conducted measurements at two specific time points during the day before breaking the fast (in the late afternoon and in the morning). To further investigate the effects of the timing of the pre-dawn meal of suhour, future studies should consider including additional time points in midday and in the evening after breaking the fast. Moreover, it’s important to note that additional time points for suhour meal consumption will be necessary for future studies to explore this research question specifically. It’s important to note that our study only involved young female athletes. Therefore, the findings observed cannot be generalized to adult males. Furthermore, the use of an auto-evaluated methodology to determine energy and macronutrient composition and sleep parameters. This approach has known weaknesses and may have played a role in the observed outcomes. It is worth noting that individual variations exist, and some athletes may adapt better to fasting during Ramadan than others, and the effectiveness of late suhoor ingestion may vary depending on factors such as an athlete’s specific sport, training schedule, and personal preferences. Personal preferences for the timing of suhoor and sleeping should be taken into account in future investigations to explore this research question specifically. Lastly, it should be noted that the absence of physiological variables, such as lactate levels, blood glucose levels, and hormones (e.g., cortisol, adrenalin, and noradrenaline), is another limitation of our current investigation. Therefore, future studies should aim to replicate our study while also monitoring and controlling these parameters.

## 5. Conclusions

The timing of suhoor, the pre-dawn meal consumed by athletes observing the fast during Ramadan, affects short-term high-intensity exercise. The findings suggest that a late suhoor, consumed closer to pre-dawn time, attenuates the decline in the performance of short-term high-intensity exercise caused by RIF, compared to early suhoor intake. The results provided further evidence for the advantageous effects of late suhoor consumption on short-term high-intensity exercise, with afternoon performance showing the greatest impact. Although the statistical findings of this study cannot be generalized, the presence of numerous significant findings and their related effect sizes could have a pair of potential implications. To begin with, adhering to Fisher’s initial statistical insights [89], the significant findings support future prospective studies in this direction. Additionally, the beneficial impacts of consuming a late suhoor meal could possibly offer a secure and efficient strategy to enhance athletes’ short-term high-intensity performance and well-being indices before the extended period of intermittent fasting during Ramadan. Considering the beneficial impact of late suhoor intake on enhancing physical performance, athletes and their coaches may explore the possibility of coordinating optimal suhoor intake strategies to align with the timing of athletic events, potentially resulting in significant performance improvements. Aiming for peak performance, this knowledge can be further leveraged to strategically plan training and competitions. Furthermore, to optimize performance during Ramadan afternoons, athletes should collaborate with dietitians or sports nutrition experts to receive personalized guidance tailored to their individual needs, training objectives, and the demands of their respective sports.

## Figures and Tables

**Table 1:** Measurement of the subjective quality of sleep recorded during 10 days before Ramadan (R − 10), Ramadan (R), and 10 days following Ramadan (R + 10).

	R − 10	R	R + 10
Sleep latency (min)	16 ± 2.4 *	17 ± 3.4 ^&^	16.6 ± 2.2
Sleep effciency (%)	95.2 ± 4.2	94.8 ± 4.2	94.4 ± 4.8
Sleep duration (h)	7.5 ± 0.8 *^#^	6 ± 0.7 ^&#^	6.6 ± 0.8 ^&^*
Sleep quality (AU)	0.5 ± 0.3 *^#^	2.2 ± 0.6 ^&#^	1.4 ± 0.6 ^&^*
Sleep disturbances (AU)	0.42 ± 0.29 *^#^	1.35 ± 0.44 ^&#^	0.9 ± 0.4 ^&^*
Daytime dysfunction (AU)	0.25 ± 0.22 *^#^	1.15 ± 0.5 ^&#^	0.59 ± 0.37 ^&^*
Total score of PSQI (AU)	2 ± 0.9 *^#^	7.2 ± 1.8 ^&#^	4.4 ± 1.9 ^&^*

AU: arbitrary units; PSQI: The Pittsburgh Sleep Quality Index; ^&^: Significant difference compared to R − 10; *: Significant difference compared to R; ^#^: Significant difference compared to R + 10.

**Table 2:** Differences in mean values ± standard deviation (SD) of body mass, body mass index (BMI), and estimated daily calories and macronutrient intake were recorded across three testing periods: 10 days before Ramadan (R − 10), 10 last days of Ramadan (R), and 10 days following Ramadan (R + 10).

	R − 10	R	R + 10	*p*-Value
Body mass (kg)	61.5 ± 6.9	61.2 ± 7	61.5 ± 7.2	0.12
Body mass index (kg/m^2^)	21.6 ± 1.8	21.5 ± 1.8	21.6 ± 1.9	0.12
Protein (g/d)	66.4 ± 12.1	64.3 ± 15.1	69.5 ± 12.4	0.31
Carbohydrate (g/d)	404.2 ± 68.3	411.4 ± 62.6	408.6 ± 63	0.13
Fat (g/d)	90.5 ± 10.4	90.8 ± 10.4	88.9 ± 10.8	0.47
Energy intake (kcal/day)	2697.3 ± 269.6	2720.4 ± 270.9	2712.9 ± 286.3	0.63

## Data Availability

The original contributions presented in the study are included in the article; further inquiries can be directed to the corresponding author.
